# Metabolic Alterations in Pancreatic Cancer Progression

**DOI:** 10.3390/cancers12010002

**Published:** 2019-12-18

**Authors:** Enza Vernucci, Jaime Abrego, Venugopal Gunda, Surendra K. Shukla, Aneesha Dasgupta, Vikrant Rai, Nina Chaika, Kyla Buettner, Alysha Illies, Fang Yu, Audrey J. Lazenby, Benjamin J. Swanson, Pankaj K. Singh

**Affiliations:** 1 The Eppley Institute for Research in Cancer and Allied Diseases, University of Nebraska Medical Center, Omaha, NE 68198, USA; enza.vernucci@gmail.com (E.V.); abrego@ohsu.edu (J.A.); venu.gunda@unmc.edu (V.G.); surendra.shukla@unmc.edu (S.K.S.); VikrantRai@creighton.edu (V.R.); nchaika@unmc.edu (N.C.); kylabuettner@gmail.com (K.B.); alyshailliescu@gmail.com (A.I.); 2 Department of Biochemistry and Molecular Biology, University of Nebraska Medical Center, Omaha, NE 68198, USA; Aneesha.dg@gmail.com; 3 Department of Biostatistics, University of Nebraska Medical Center, Omaha, Nebraska, NE 68198, USA; fangyu@unmc.edu; 4 Department of Pathology and Microbiology, University of Nebraska Medical Center, Omaha, NE 68198, USA; alazenby@unmc.edu (A.J.L.); bjswanso@unmc.edu (B.J.S.); 5 Department of Genetics Cell Biology and Anatomy, University of Nebraska Medical Center, Omaha, NE 68198, USA

**Keywords:** pancreatic cancer, precancerous lesions, cancer metabolism, metabolic alterations, KPC mice

## Abstract

Pancreatic cancer is the third leading cause of cancer-related deaths in the USA. Pancreatic tumors are characterized by enhanced glycolytic metabolism promoted by a hypoxic tumor microenvironment and a resultant acidic milieu. The metabolic reprogramming allows cancer cells to survive hostile microenvironments. Through the analysis of the principal metabolic pathways, we identified the specific metabolites that are altered during pancreatic cancer progression in the spontaneous progression (KPC) mouse model. Genetically engineered mice exhibited metabolic alterations during PanINs formation, even before the tumor development. To account for other cells in the tumor microenvironment and to focus on metabolic adaptations concerning tumorigenic cells only, we compared the metabolic profile of KPC and orthotopic tumors with those obtained from KPC-tumor derived cell lines. We observed significant upregulation of glycolysis and the pentose phosphate pathway metabolites even at the early stages of pathogenesis. Other biosynthetic pathways also demonstrated a few common perturbations. While some of the metabolic changes in tumor cells are not detectable in orthotopic and spontaneous tumors, a significant number of tumor cell-intrinsic metabolic alterations are readily detectable in the animal models. Overall, we identified that metabolic alterations in precancerous lesions are maintained during cancer development and are largely mirrored by cancer cells in culture conditions.

## 1. Introduction

Genetic instability plays a pivotal role in pancreatic ductal adenocarcinoma (PDAC) initiation and progression [1]. Somatic alterations in PDAC, including chromosomal copy-number gains, amplifications, loss of heterozygosity, deletions, and point mutations have been characterized previously [2,3]. Activating mutations in KRAS are the major drivers for PDAC initiation and are present even in low-grade pancreatic intraepithelial neoplasia (PanIN) [4]. PanINs begin as low-grade lesions that, over time, allow premalignant cells to accumulate mutations. These alterations promote genetic instability and tissue disorganization, thus allowing histological characterization of PDAC progression from low-grade PanIN lesions to high-grade PanIN lesions in a stepwise manner before the formation of an invasive tumor mass [5]. Several studies have demonstrated that mutations in Kras alone are sufficient to initiate PanINs, but not enough to develop an invasive form of pancreatic cancer [6,7]. The cooperation between activated KRAS and inactivated tumor suppressor genes is required for progression of the disease. TP53 is one of the suppressor genes most frequently altered in pancreatic cancer [8]. While deletions in p53 are frequently observed in PDAC, many PDAC tumors carry gain-of-function mutations in PDAC that make tumors more aggressive and metastatic [7]. Both, KRAS and TP53 are known to regulate metabolic reprogramming in cancer [9,10]. On the one hand, activating Kras mutations support tumor growth by enhancing glucose uptake and directing glucose carbon flux into biosynthetic pathways such as the pentose phosphate pathway (PPP). Furthermore, Kras-driven pancreatic cell lines increase glutamine dependency once exposed to low pH, a cancer hallmark [11]. TP53, on the other hand, is known to regulate glycolysis, PPP, and oxidative phosphorylation [10].

Pancreatic cancer patients demonstrate a five-year survival rate of around 8%, one of the lowest across all cancer types [12]. Gemcitabine, alone or in combination with other drugs, is still the first line of therapy for pancreatic cancer. However, patients often develop resistance to the drug, reducing the effectiveness of treatment [13]. In the last decade, very few achievements have been reached to improve the quality of life and the survival rates of pancreatic cancer patients. The development of new cancer drugs relies on animal models to validate the efficacy of novel therapies. Therefore, it is crucial to corroborate and characterize PDAC models. Subcutaneous tumor models present easy surgical feasibility and affordable cost for extensive drug screening even if they seldom manifest metastasis and the appropriate tumor microenvironment [14]. Orthotopic models mirror tumorigenicity and metastasis incidence in patients but can present greater perfusion, which impacts drug delivery [15]. Researchers have utilized different genetically engineered mouse models to study PanINs and the invasive form of pancreatic cancer. The Pdx1-Cre; LSL-KrasG12D (KC) mouse model [16] that expresses activating KrasG12D mutation in the pancreas is an excellent model to study the early progression of pancreatic tumors. Early PanINs are visible in two-week-old mice, and malignant transformation of high-grade lesions positively correlates with the age of the mice in the KC model [16]. The low-frequency and slow onset of invasive disease accentuated the need to develop new models able to reproduce the features of human pancreatic cancer more effectively. To recapitulate the aggressive disease, the KC model was further modified to carry alterations or deletions in tumor suppressors, including p53 [7], SMAD4 [17], and p16 [6]. The model harboring PDX1-Cre; LSL-KrasG12D; p53R172H, otherwise known as KPC, was developed by Hingorani and colleagues [7]. The KPC model is the most extensively utilized PDAC model for understanding the molecular mechanisms driving the disease. KPC mice tumors recapitulate most of the peculiarities of human pancreatic cancer, including poor vascularization, moderately differentiated pancreatic ducts, resistance to gemcitabine chemotherapy, and predominant metastasis to liver and lungs [15].

Altered metabolism is one of the hallmarks of cancer [18]. In 1920 Otto Warburg discovered that cancer cells use aerobic glycolysis to increase biomass production to sustain cell proliferation at the expense of ATP production [19]. Subsequently, it has been shown that cancer cells reroute glucose and other nutrients into anabolic pathways to provide building blocks essential for cell division [20]. Multiple oncogenes and signaling pathways, beyond Kras and p53, have since been demonstrated to regulate metabolic reprogramming and contribute to poor therapy response [20,21,22,23,24]. As metabolic reprogramming plays a pivotal role in cancer initiation, progression, and therapy responsiveness, different diagnostic techniques based on metabolism are routinely utilized in the clinic. For example, increased glucose uptake in tumor cells allows the use of radiolabeled glucose analog 18F-fluorodeoxyglucose and positron emission tomography (FDG-PET) to analyze tumor burden in a noninvasive way. Imaging exchange using hyperpolarized [1–13C] pyruvate [25] has been utilized to follow the progression of preneoplastic pancreatic lesions for diagnostic purposes in KrasG12D–based models. Given the importance of metabolism for detecting and monitoring pancreatic tumors, a complete metabolic characterization of the precancerous lesions and invasive tumors can be extremely useful for early tumor detection.

Interestingly, desmoplasia represents a peculiar characteristic of pancreatic adenocarcinoma since the fibrous stroma, including cellular and noncellular components, surrounding the tumor can reach up to 90% of the tumor volume [26]. Thus, in a scenario where the desmoplastic reaction can be so massive, it can become difficult to analyze the metabolic behavior of cancer cells alone. In this study, through a multi-pronged approach, using the PDAC murine models that better recapitulate the human disease, we show the metabolic changes that occur in vivo starting from early PanINs to invasive cancer. To reduce the stromal component contribution in metabolic perturbations, we compared our findings with in vitro models to detect tumor-specific metabolic alterations that drive cancer development.

## 2. Results

### 2.1. Pathological Evaluation of the Pancreas from KPC Mice at Different Stages

**Figure 1:**
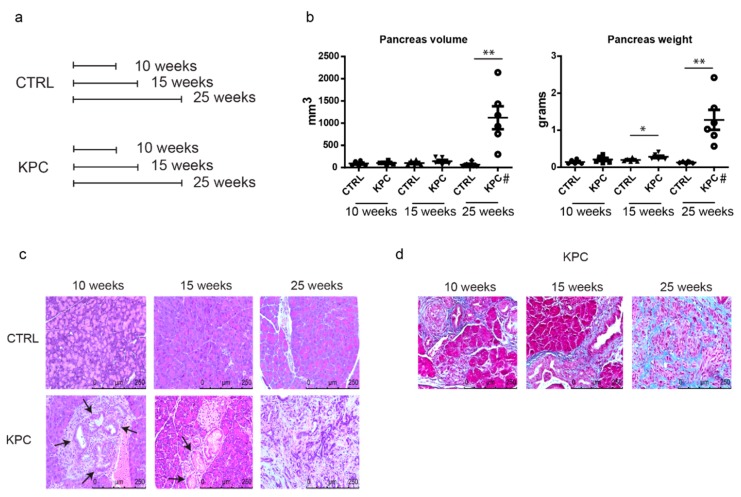
Pathological evaluation of the pancreas from KPC mice at different stages. (**a**) Scheme of the experimental design. Six mice per group with the appropriate genotype were sacrificed after 10, 15, and 25 weeks. (**b**) Graphs representing tumor volumes and weights at the time of necropsy Tissues volume and weight were compared by Student’s t-test. ‘*’ *p* < 0.05 and ‘**’ *p* < 0.01. # indicates the group where pancreatic tumors were found. (**c**) Hematoxylin and eosin stains in pancreas/tumor sections from control and KPC mice. Control mice at 10 weeks, 15 weeks, and 25 weeks showed normal acinar architecture in the pancreas with no PanIN lesions and no adenocarcinoma (top row). KPC mice pancreas showed PanIN lesions (indicated by arrowheads) at 10 and 15 weeks and invasive adenocarcinoma at 25 weeks (bottom row). (**d**) Masson’s trichrome staining in pancreas/tumor sections from KPC mice for evaluating fibrosis and desmoplastic reaction. Fibrosis/desmoplastic regions are stained blue. Normal periductal fibrous tissue but not significant desmoplasia can be noticed at 10 and 15 weeks in KPC mice. At 25 weeks, the invasive adenocarcinoma demonstrates tumor-associated desmoplasia.

To analyze metabolic and enzymatic changes that happen during pancreatic cancer development, we used the KPC mouse model. Each group, comprising of six mice, was sacrificed 10, 15, and 25 weeks after birth (Figure 1a). Post-necropsy, pancreas volume, and weight were measured (Figure 1b). The pancreas collected from KPC after 10 weeks weighed similar to the non-KPC littermate controls. However, the pancreas from KPC mice at 15 weeks after birth showed a significant increase in weight, probably due to inflammation, a phenomenon also seen in patients with pancreatitis [27]. The KPC mice at 25 weeks presented tumors. The histological evaluation was also performed to highlight differences between groups. No changes were detected across the pancreas obtained from control groups collected from 10, 15, and 25-week-old mice. The acini, the islets of Langerhans cells, and pancreatic ducts preserved their normal structure. PanINs were evaluated in the pancreas from 10- and 15-week-old KPC mice to estimate the histological grade of precursor lesions. Ducts with characteristics attributable to low-grade and high-grade PanINs were observed in both groups. Columnar cells with nuclei located at the base and lesions consisting of the loss of nuclear polarity were present (Figure 1c). The 25-week-old KPC group was the only one that was characterized by tumor onset and the development of desmoplastic reaction (Figure 1d).

### 2.2. Evaluation of Glycolysis Pathways in Pancreatic Cancer Models

**Figure 2:**
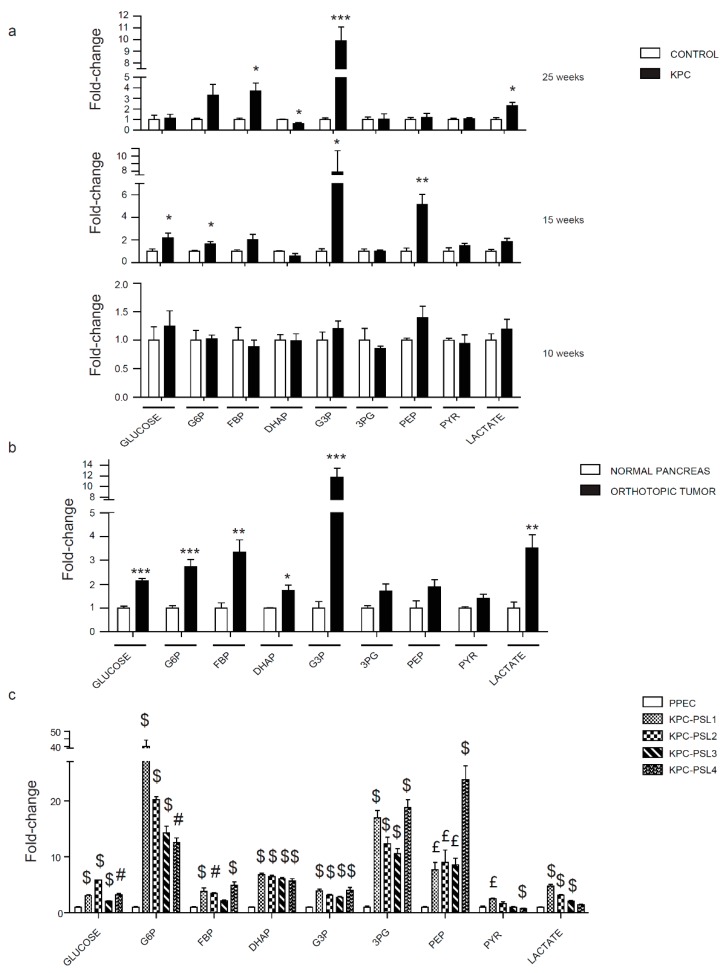
Evaluation of glycolysis metabolites in pancreatic cancer models. (**a**) Relative glycolytic metabolite levels in 10, 15, and 25 weeks old KPC mice pancreas/tumors determined by LC-MS/MS. (**b**) Relative glycolytic metabolite levels in mice orthotopically implanted with a KPC cell line. (**c**) Relative glycolytic metabolite levels in KPC cell lines. Abbreviations: G6P, glucose-6-phosphate; FBP, fructose bisphosphate; DHAP, dihydroxyacetone phosphate; G3P, glyceraldehyde-3-phosphate; 3PG, 3-phosphoglycerate; PEP, phosphoenolpyruvate; PYR, pyruvate. Data are represented as mean ± SEM. The bar charts in (**a**) and (**b**) were compared by Student’s t-test. * *p* < 0.05, ** *p* < 0.01, and *** *p* < 0.001. Bar charts in (**c**) were compared by one-way ANOVA followed by Tukey’s post-hoc test. £ *p* < 0.05, # *p* < 0.01, and $ *p* < 0.001.

Increased glycolysis is a phenotype observed in most human cancers. Tumorigenic cells increase the uptake of glucose and other nutrients to fuel uncontrolled cell proliferation [28]. To investigate the status of glycolysis, we investigated the expression levels of glycolytic genes and metabolites. Glucose is transported inside the cells through an energy-independent mechanism mediated by glucose transporters, GLUTs [29]. GLUT1 is a highly expressed transporter in most cancers. The expression of GLUT1 in pancreatic cancer correlates with tumor size, higher stage, and metastasis in lymph nodes [30]. Interestingly, in our analysis, glucose did not show significant variation during tumor progression, perhaps due to rapid metabolism in tumors (Figure 2a). The metabolic profile did not show any variation between KPC and control mice at 10 weeks. At 15 weeks, glucose-6-phosphate, glyceraldehyde-3-phosphate, and phosphoenolpyruvate were significantly increased in KPC pancreas (Figure 2a). At 25 weeks when the tumor was already formed, a remarkable increase in fructose-1,6-bisphosphate, glyceraldehyde-3-phosphate, and lactate was observed; dihydroxyacetone phosphate was the only metabolite that showed a significant reduction (Figure 2a). To verify the consistency of our data in the KPC model, an orthotopic mouse model using a KPC cell line, was utilized for metabolomic comparison. Upon comparison of healthy pancreas and tumor-bearing mice, we observed significant upregulation of glucose, glucose-6-phosphate, fructose-1,6-bisphosphate, dihydroxyacetone phosphate, glyceraldehyde-3-phosphate, and lactate in pancreatic tumors (Figure 2b). We also performed in vitro experiments to assess whether our results represented a combinatorial effect due to contributions from other cell types or a reflection of the metabolic behavior of cancer cells. Analysis of glycolytic metabolites in KPC cell lines revealed an upregulation in all the metabolites in the pathway as compared with the immortalized mouse pancreatic epithelial cell line (Figure 2c). Altogether, these results describe increased glycolysis during tumor progression in the KPC, orthotopic, and in vitro models.

### 2.3. Evaluation of the Pentose Phosphate Pathway in Pancreatic Cancer Models

**Figure 3:**
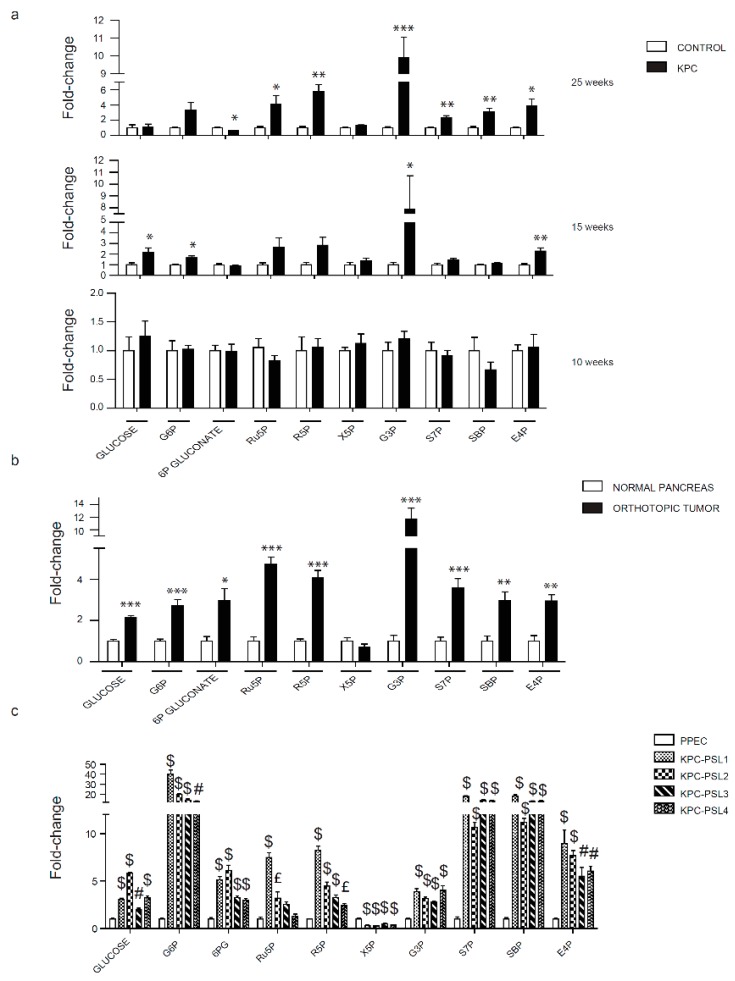
Evaluation of the pentose phosphate pathway metabolites in pancreatic cancer models. (**a**) Relative PPP metabolite levels in 10, 15, and 25 weeks old KPC mice. (**b**) Relative PPP metabolite levels in orthotopically implanted mice. (**c**) Relative PPP metabolite levels in KPC cell lines. Abbreviations: G6P, glucose-6-phosphate; Ru5P, ribulose-5-phosphate; R5P, ribose-5-phosphate; X5P, xylulose-5-phosphate; G3P, glyceraldehyde-3-phosphate; S7P, sedoheptulose-7-phosphate; SBP, sedoheptulose 1,7-bisphosphate; E4P, erythrose 4-phosphate. Data are represented as mean ± SEM. The bar charts in (**a**) and (**b**) were compared by Student’s *t*-test. * *p* < 0.05, ** *p* < 0.01, and *** *p* < 0.001. Bar charts in (**c**) were compared by one-way ANOVA followed by Tukey’s post-hoc test. £ *p* < 0.05, # *p* < 0.01, and $ *p* < 0.001.

PPP is one of the most highly regulated pathways in cancer cells [31]. The importance of PPP is evident from its role in modulating two critical pathways. First, it is involved in ROS regulation through NADPH production, and then, it converts glucose to ribose-5-phosphate, which is indispensable for nucleotide biosynthesis. Evaluation of glycolysis indicated an increase in glucose-6-phosphate and glyceraldehyde-3-phosphate, metabolites that are in common between the two pathways. Thus, we next evaluated the levels of metabolites in the PPP. Metabolomic analysis indicated no change in the PPP metabolites between the pancreas of KPC and the control mice at 10 weeks. Five weeks later, a significant increase in glucose, glucose-6-phosphate, erythrose-4-phosphate, and glycerol-3-phosphate was detected. At 25 weeks, all the metabolites except for glucose, glucose-6-phosphate, and xylulose-5-phosphate, demonstrated a significant increase (Figure 3a). The metabolic analysis of orthotopically implanted KPC cell line-derived tumors showed an upregulation of the majority of the metabolites as compared with the pancreas from the control mice. Xylulose-5-phosphate was the only metabolite that did not show any alteration (Figure 3b). An analysis of PPP metabolites in cells in culture indicated a substantial increase in the pathway metabolites in all the cell lines, except xylulose-5-phosphate, which was significantly downregulated (Figure 3c). Altogether, in vivo and in vitro results showed an overall upregulation of PPP.

### 2.4. Evaluation of the TCA Cycle in Pancreatic Cancer Models

**Figure 4:**
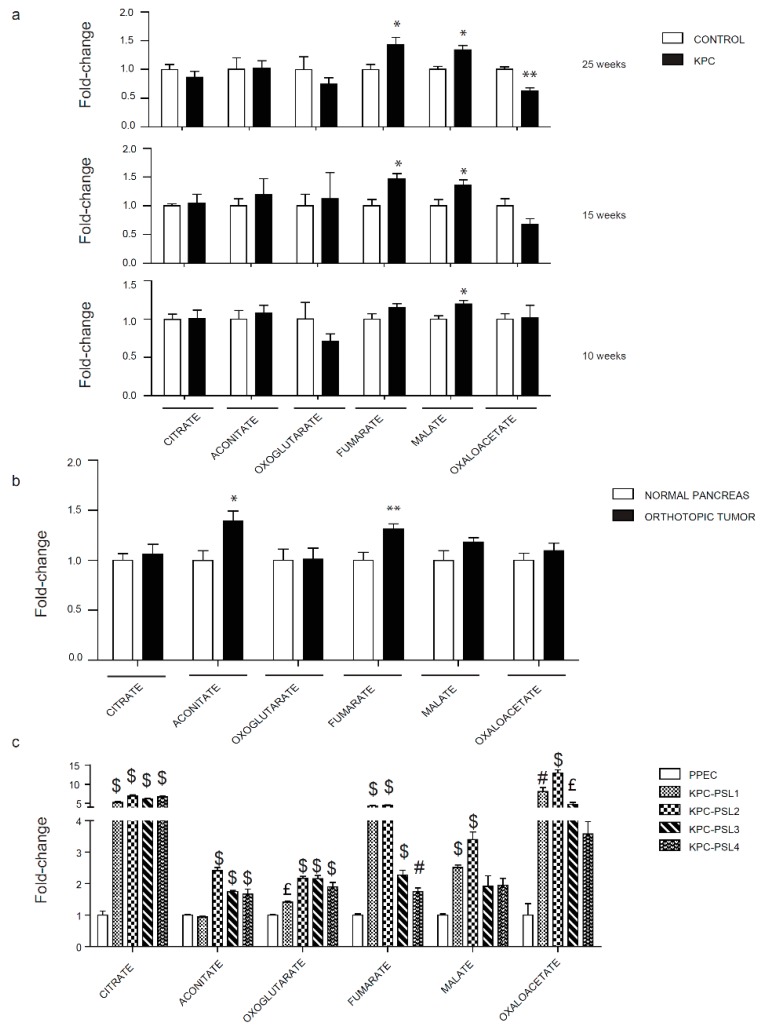
Evaluation of the TCA cycle metabolites in pancreatic cancer models. (**a**) Relative TCA cycle metabolite levels in 10, 15, and 25 weeks old KPC mice. (**b**) Relative TCA metabolite levels in orthotopically implanted mice. (**c**) Relative TCA metabolite levels in KPC cell lines. Data are represented as mean ± SEM. The bar charts in (**a**) and (**b**) were compared by Student’s t-test. * *p* < 0.05 and ** *p* < 0.01. Bar charts in (**c**) were compared by one-way ANOVA followed by Tukey’s post-hoc test. £ *p* < 0.05, # *p* < 0.01, and $ *p* < 0.001.

The TCA cycle is the central route for energy metabolism, redox maintenance, and biomolecule synthesis. Glucose, glutamine, and fatty acids can be channeled into the TCA cycle and provide nutrients to meet the metabolic needs of the tumor. The critical role of the TCA cycle in tumorigenesis led us to investigate metabolic changes in the TCA cycle in our models. We observed alterations in a few metabolites in the spontaneous model. Only malate was consistently upregulated at 10, 15, and 25 weeks (Figure 4a). Fumarate increased only at 15 and 25 weeks, whereas oxaloacetate decreased at 25 weeks (Figure 4a). The metabolic profile of the tumors generated from the KPC cell line indicated increased levels of aconitate and fumarate (Figure 4b). A substantial increase was detected in multiple TCA cycle metabolites using four KPC cell lines under culture conditions (Figure 4c).

### 2.5. Evaluation of Purine and Pyrimidine Biosynthesis Pathways in Pancreatic Cancer Models

**Figure 5:**
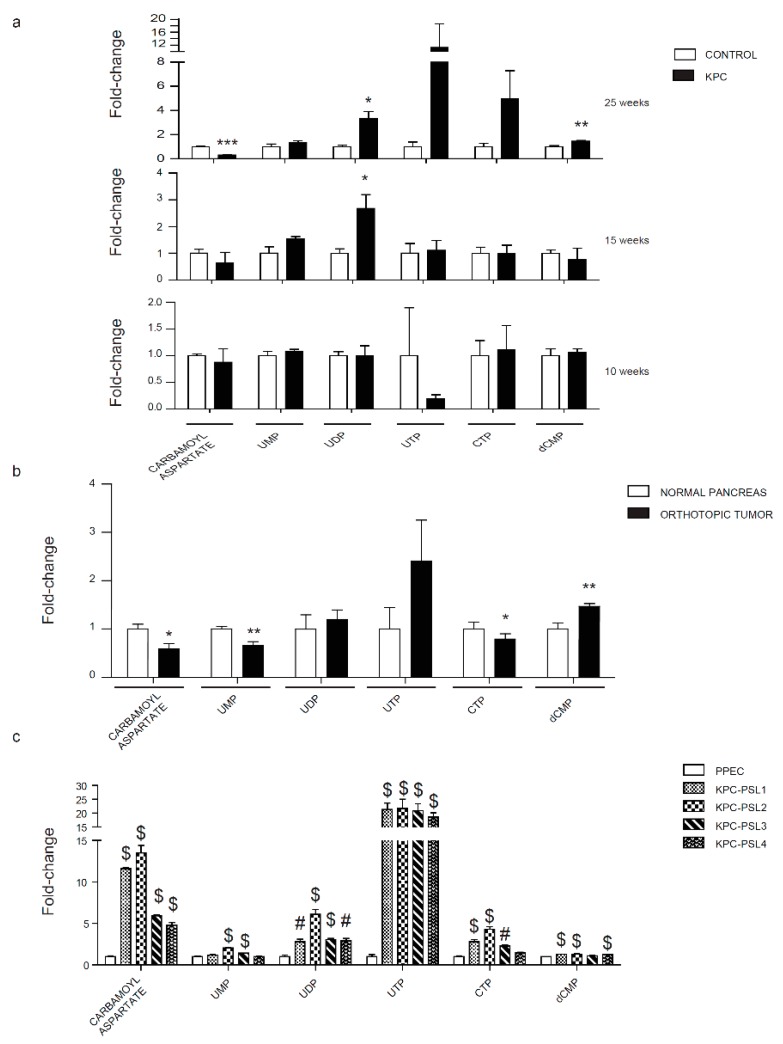
Evaluation of the pyrimidine pathway in pancreatic cancer models. (**a**) Relative pyrimidine metabolite levels in 10, 15, and 25 weeks old KPC mice. (**b**) Relative pyrimidine metabolite levels in orthotopically implanted mice. (**c**) Relative pyrimidine metabolite levels in KPC cell lines. Abbreviations: UMP uridine 5’-monophosphate, UDP uridine 5’-diphosphate, UTP uridine-5’-triphosphate, dCMP deoxycytidine 5’-monophosphate, CTP cytidine-5’-triphosphate. Data are represented as mean ± SEM. The bar charts in (**a**) and (**b**) were compared by Student’s t test. * *p* < 0.05, ** *p* < 0.01, and ***p < 0.001. Bar chart in (**c**) was compared by one-way ANOVA followed by Tukey’s post-hoc test. # < 0.01 and $ < 0.001.

Purines and pyrimidines are required for DNA and RNA synthesis that are essential for the continuous growth of cancer cells. Nucleotides analogs, including gemcitabine and 5-FU, are still utilized in the clinic to inhibit aberrant cancer cell proliferation [32]. Interestingly, we did not observe any significant increase in purine biosynthesis pathway metabolites during cancer progression. dAMP, GMP, and dGMP showed a significant decrease as compared with the control in 15 and 25 weeks (Supplementary Figure S1a). We also observed GMP reduction in tumors in the orthotopic model (Supplementary Figure S1b). We also noted increasing trends for ADP and ATP levels in spontaneous and orthotopic models and significant upregulation in all the KPC cell lines (Supplementary Figure S1c). Consistent with the in vivo results, GMP was found to be downregulated in the cell culture models as well (Supplementary Figure S1c). Evaluation of the pyrimidine pathway, in contrast, revealed an increase in UDP and dCMP levels and a decrease in carbamoyl-aspartate levels during pancreatic cancer progression (Figure 5a). We noted an increase in dCMP and CTP, and downregulation of carbamoyl-aspartate and UMP levels in the orthotopic model (Figure 5b). An overall increase in all the pyrimidine pathway metabolites was detected in KPC cell lines (Figure 5c).

### 2.6. Evaluation of Urea Cycle and Amino Acids in Pancreatic Cancer Models

**Figure 6:**
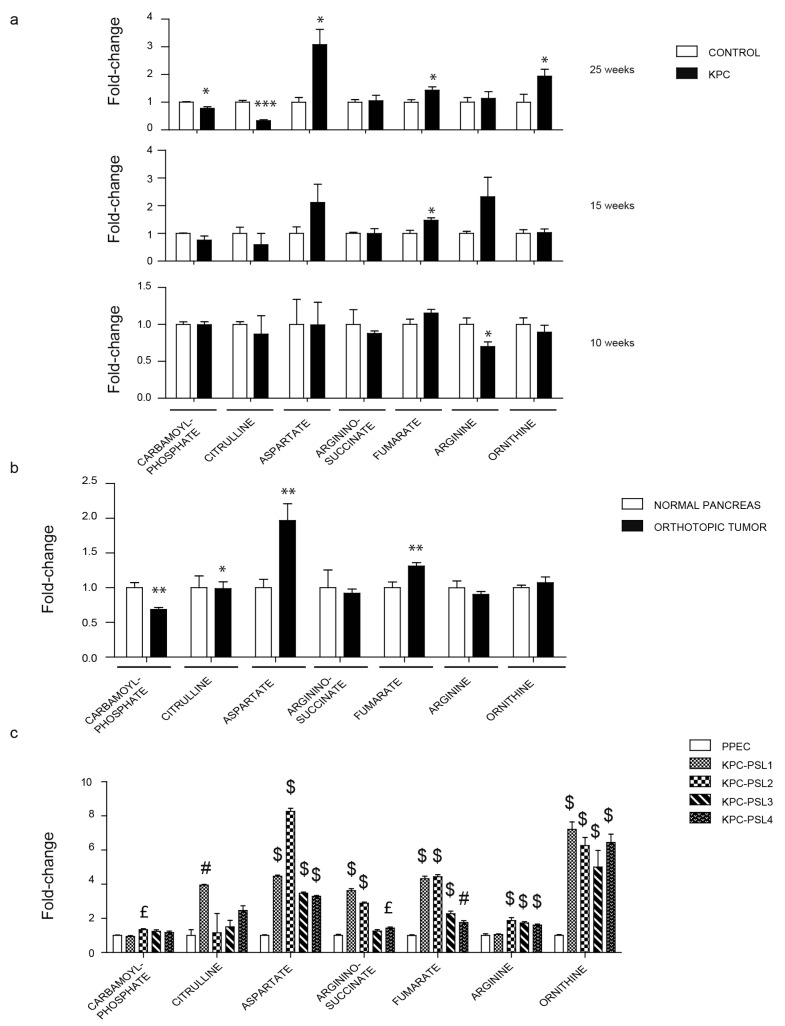
Evaluation of the urea cycle metabolites in pancreatic cancer models. (**a**) Relative urea cycle metabolite levels in 10, 15, and 25 weeks old KPC mice. (**b**) Relative urea cycle metabolite levels in orthotopically implanted mice. (**c**) Relative urea metabolite levels in KPC cell lines. Data are represented as mean ± SEM. The bar charts in (**a**) and (**b**) were compared by Student’s t-test. * *p* < 0.05, ** *p* < 0.01, and *** *p* < 0.001. Bar charts in (**c**) were compared by one-way ANOVA followed by Tukey’s post-hoc test. £ *p* < 0.05, # *p* < 0.01, and $ *p* < 0.001.

Aspartate is known to play a significant role in nucleotide biosynthesis that, in turn, supports cancer growth. Aspartate is a substrate for disparate enzymes, including argininosuccinate synthase that is involved in the urea cycle. The urea cycle is crucial for transferring nitrogen from ammonia and aspartate to urea. In our study, during pancreatic cancer progression, we noticed a significant decrease in arginine in 10-week-old mice. We observed a reduction in carbamoyl-phosphate and citrulline, and an upregulation of aspartate, fumarate, and ornithine in tumors from 25-week-old KPC mice (Figure 6a). We observed decreased carbamoyl-phosphate and increased aspartate and fumarate levels in the orthotopic model (Figure 6b). Citrulline was the only metabolite showing an opposite trend among the in vivo models (Figure 6b). The metabolic analysis of KPC cell lines revealed a substantial increase in aspartate, fumarate, and ornithine in all the cell lines, while argininosuccinate and arginine levels were upregulated in most of the KPC cell lines (Figure 6b). We also analyzed amino acid levels in our experimental models. We noted an increase in serine and a decrease in arginine levels in 10-week-old KPC mice. Valine, leucine, and tryptophan were upregulated in the pancreas from 15-week-old KPC mice as compared with the controls. In 25-week-old KPC mice, levels of valine, tryptophan, lysine, and aspartate were upregulated, while tyrosine and cysteine showed a significant downregulation (Supplementary Figure S2a). In the orthotopic model, we observed increased serine, asparagine, cysteine, and aspartate, but tyrosine and glutamate levels were reduced as compared with the control mice (Supplementary Figure S2b). The amino acids were significantly increased in all the KPC cell lines as compared with the immortalized mouse pancreatic epithelial cell line (Supplementary Figure S2c).

## 3. Discussion

Cancer cells undergo metabolic reprogramming to adapt to the proliferative demands and stressful microenvironments by increasing the uptake of nutrients and their incorporation in the biosynthetic pathways [22,33,34]. The Warburg effect represents metabolic adaptations in proliferating cells and cancer cells where increased glucose uptake, despite the presence of oxygen, sustains anabolic pathways that culminate into biomass production. In this study, we demonstrate that cancer cells undergo a wide array of metabolic changes that occur in parallel to the tumor progression. This study highlights the fact that normal cells destined to become cancerous start hijacking and rearranging metabolism for their advantage even when the malignant phenotype is not yet visible. In fact, at 15 weeks and, in some cases, even at 10 weeks, alterations in the principal metabolic pathways can be appreciated even when only PanIN lesions are present (Figure 1c).

Serum-based metabolic markers have been identified to discriminate between benign disease and pancreatic cancer [35]. Using proton nuclear magnetic resonance spectroscopy, it has been shown that cancer patient serum carries higher glutamate and glucose levels, whereas creatinine and glutamine are elevated in benign cases [35]. A different study detected a high level of branched-chain amino acids in plasma samples associated with pancreatic cancer patients [36]. Although useful from the diagnostic point of view, the connection with the tumor metabolic phenotype remains unclear. In our study, we evaluated the metabolic changes in the tumors and in cell lines that occur during cancer development and growth (Supplementary Figures S3 and S4).

The tumor microenvironment also governs the overall aggressive behavior of tumors. It is known that signaling interactions between stroma components and pancreatic cancer cells assist and support tumor growth [37]. The tumor microenvironment consists of several cells, including pancreatic stellate cells, tumor-associated macrophages, neutrophils, and T cells. Therefore, being able to discriminate and understand the complex crosstalk between all the components has been challenging for researchers. We believe our study adds a piece to this puzzle; the comparison between animal models and culture conditions, in fact, identifies metabolic markers related only to cancer cells that can represent promising targets for future therapies. Of note, some metabolites showed different trends across the experimental models. Carbamoyl-aspartate levels, an essential precursor to pyrimidines, showed an increase in KPC-derived lines, whereas it decreased in 25-week-old KPC tumors. While such results could, in part, be due to extensive stromal contributions, artifacts of cell culture can lead to similar discrepancies. Nonetheless, metabolites with a dual behavior cannot be used as potential tumor biomarkers.

Kras and p53 are key metabolic regulators, and, according to the TCGA database, 90% and 70% of pancreatic cancer patients have alterations in these two genes, respectively. Our study is the first to demonstrate the role of Kras and p53 in driving metabolic changes even before the occurrence of malignant transformation. Different stages of tumor development have been investigated; 10 and 15-week-old groups represent two steps that precede tumor formation, where only PanINs are present and the 25-week-old group is characterized by the metastatic disease. Glycolysis and PPP are the two pathways that showed an overall significant increase (Figure 2, Figure 3). Fructose bisphosphate, glyceraldehyde-3-phosphate, and lactate are the three metabolites unanimously upregulated in our three different experimental models. ALDOA, the gene encoding the glycolysis enzyme that converts fructose-1,6-bisphosphate to glyceraldehyde-3-phosphate, has been shown to correlate with poor prognosis in pancreatic cancer patients [38]. LDHA, which catalyzes the last step of glycolysis, is upregulated in pancreatic cancer specimens [39]. Since serum lactate dehydrogenase increases during tissue damage or disease [40], an elevated concentration of lactate in serum has been used in several cancers as a prognostic marker for poor survival [41]. Although additional studies are needed for pancreatic cancer, our study showed that there is a progressive increase in lactate accumulation inside the cells during tumor progression that is linked to an increase in LDHA expression in pancreatic cancer. The PPP demonstrated the highest number of altered metabolites during screening in mice models and in cell lines in culture. This is in accordance with what has been already shown about Kras and its role in reprogramming cell metabolism in PDAC [9]. Using mice expressing Kras G12D under the control of a tet-operator and a lox-stop-lox cassette, Haoqiang Ying et al. showed that Kras inhibition can reduce glucose uptake, O-linked N-acetylglucosamine posttranslational modification, and decrease the flux into PPP with consequent reduction in ribose production essential for nucleotides biosynthesis [42]. In our analysis, ribose -5-phosphate was remarkably increased, thereby indicating the importance of the PPP for DNA synthesis in cancer cells. In the TCA cycle, fumarate was the only common polar metabolite upregulated in our study. Fumarate has been reported to play an important role in stabilizing HIF-1 alpha [43], one of the major drivers of the metabolic shift. Our study brought to light the strong alteration that characterizes this metabolite, starting from the formation of PanINs. Considering that we also noted significant increases in aspartate, at late stage KPC lesions, and malate, even at early stage KPC lesions, the metabolic changes could be suggestive of anaplerotic glutamine metabolism that facilitates tumor cell survival under low pH [11]. While we noted similar trends, purines and pyrimidines did not show a statistically significant amplification in mice models as compared with KPC cells. It is possible that the complex heterogeneity of the tumor microenvironment dilutes the changes in these two biosynthetic pathways known to be strongly upregulated in cancer cells. Alternatively, decreased levels can also be indicative of high consumption rates that can be confirmed with additional kinetic flux studies. Furthermore, heterogeneity of tumor progression/growth may require more replicates to achieve statistical significance. Recent studies demonstrate a correlation between urea cycle alteration and cancer cell proliferation, where expression of argininosuccinate synthase (ASS1) correlates with the amount of aspartate channeled into pyrimidine pathway to fuel cancer growth [44]. While we did not investigate ASS1 expression, our results demonstrate elevated aspartate levels. However, no concordant changes are detected for citrulline and argininosuccinate in our models. The levels of the other amino acids differ among the two in vivo models. Macropinocytosis plays a role in the uptake of external nutrients subsequently degraded in lysosomes. It is a phenomenon activated by mutant Kras, which is a key driver for pancreatic cancer progression [45]. Differences in degrees of blood perfusion between KPC and the orthotopic model could in part be responsible for the amino acid level variations in the tumors.

Several studies have highlighted the connection between aberrant metabolism and increased drug resistance. In pancreatic cancer, the standard of care is gemcitabine, a deoxycytidine analog, used alone or in combination with other drugs. The capacity of cancer cells to develop resistance to this drug in vivo and in vitro demonstrates the urgency to understand the mechanisms that gives rise to gemcitabine resistance. Several hypotheses have been proposed about the advantages that alterations in metabolism can confer to cancer cells. Our lab demonstrated that by increasing glucose uptake and metabolic flux through the non-oxidative arm of pentose phosphate pathway, gemcitabine-resistant cancer cells increase pyrimidine synthesis which competes with gemcitabine incorporation [20]. Furthermore, we showed how pancreatic cancer relies on fatty acids biosynthesis for gemcitabine resistance [46]. A different approach to reduce gemcitabine resistance is through a glutamine analog. In fact, a recent study demonstrated that the compound 6-diazo-5-oxo-L-norleucine, a glutamine analog, can affect the hexosamine biosynthesis pathway, reducing the protein glycosylation, and impacting the function of several receptor tyrosine kinases such as EGFR [47].

Overall our study demonstrates metabolic reprogramming in early lesions of pancreatic cancer progression characterized by an increase in glycolysis and pentose phosphate pathway. Furthermore, a number of metabolites in the TCA cycle, urea cycle, anaplerotic glutamine metabolism, and nucleotide biosynthesis pathway were induced across multiple models. The comparative analysis of different models can be advantageous for discriminating cancer markers that are less affected by the tumor microenvironment or artifacts of in vitro settings.

## 4. Materials and Methods

### 4.1. In Vivo Experiments

All the in vivo studies were approved by the University of Nebraska Medical Center’s Institutional Animal Care and Use Committee (IACUC) (protocol approval number: 19-046-05-FC). KPC mice were bred and randomly divided into three groups of six mice each. Pancreas and tumors were harvested after 10, 15, and 25 weeks; weights and volumes were evaluated. C57 albino mice were bred in-house and 8-week-old mice were implanted orthotopically. Then, 5 × 10^5^ cells, from two different KPC cell lines, were implanted in each mouse. One week after implantation, mice were monitored and were euthanized once the tumor burden reached 1 cm^3^ according to the “Guide for the Care and Use of Laboratory Animals” [48].

### 4.2. Cell Isolation from KPC Tumor

Tumors were collected under sterile conditions and harvested in Dulbecco’s modified Eagle medium (Hyclone, Waltham, MA, USA) supplemented with 20% of FBS (Fisherbrand, Waltham, MA, USA), penicillin-streptomycin, and Antibiotic-Antimycotic (Gibco, Waltham, MA, USA). Immediately, tumors were cut into small pieces, washed twice with 1X PBS, resuspended in 10 mL of 1X PBS containing Trypsin (Hyclone, Waltham, MA, USA), Collagenase IV (Gibco, Waltham, MA, USA) and CaCl2, and incubated at 37 °C for 1 h. Tumor pieces were washed twice in 1X PBS and placed in a 10 cm dish completely covered with DMEM including 10% FBS and Antibiotic-Antimycotic in the incubator. Once 80% of cellular confluence was reached, tumor pieces were removed, and the cells were trypsinized. The first cells to detach were isolated, and the remaining cells were further trypsinized and seeded at a very low density to reduce fibroblast contamination. Alpha-SMA antibody was used to show the absence of fibroblast contamination. The KPC cell lines (KPC-PSL1, KPC-PSL2, KPC-PSL3, KPC-PSL4) were authenticated by sequencing after performing PCR using the following primers: Kras forward 5′-ACTTGTGGTGGTTGGAGCTG-3′, Kras reverse 5′-TGACCTGCTGTGTCGAGAAT-3′, P53 forward 5′-CACGTACTCTCCTCCCCTCA-3′, and P53 reverse 5′-ATTTCCTTCCACCCGGATAA-3′. Obtained cell lines were characterized, and their tumorigenic potential was tested after 40 passages in cell culture. All the KPC cell lines were tested for Mycoplasma contamination, and only Mycoplasma-free cells were utilized for any assays. In all the experiments, KPC cell lines were compared to immortalized mouse pancreatic epithelial cell line (PPEC) obtained from ATCC.

### 4.3. Metabolomic Analysis of Tumor and Cell Lines

Frozen pancreatic tissue samples were weighed and homogenized on dry ice using a sonicator (Fisher Scientific 550 sonic dismembrator). Cells were sonicated using the Continuous Mode (5s × three times) using a frequency of 20 kHz and metabolites were extracted with 80% ice-cold methanol [49]. KPC cell lines were seeded at 70% confluence and cultured for 24 h. Two hours before harvesting, culture media was changed with fresh media. Cells were rinsed with PBS and frozen on dry ice for metabolite extraction and analysis, as previously reported [49,50].

### 4.4. Immunohistochemistry

Tissues were fixed in 10% formalin for 24 h and embedded in paraffin. Sections, 5 µm thick, were deparaffinized, rehydrated progressively, and stained with Gill’s Hematoxylin (Sigma-Aldrich, St Louis, MO, USA) and Eosin Y solution (Fisher Scientific, Waltham, MA, USA) according to the manufacturer’s guidelines. Masson’s trichrome staining (MTS) was performed according to the manufacturer’s instructions (Sigma-Aldrich). Number and stage of PanINs, as well as the desmoplastic reaction, were evaluated by a board–certified pathologist in a blinded manner.

## 5. Conclusions

Our results delineate the cellular metabolism reprogramming that starts early in PDAC progression and demonstrate that such changes are consistently observed across different models. While some of the metabolic changes in tumor cells are not detectable in orthotopic and spontaneous tumors, a significant number of tumor cell-intrinsic metabolic alterations are readily detectable in the animal models. Hence, our studies provide novel insights into the metabolic reprogramming in PDAC and could facilitate the identification of novel therapeutic targets or biomarkers for the initial steps in cancer development.

## Supplementary Materials

The following are available online at https://www.mdpi.com/2072-6694/12/1/2/s1, Figure S1: Evaluation of pyrimidine pathway in pancreatic cancer models, Figure S2: Evaluation of amino acids levels in pancreatic cancer models, Figure S3: Metabolic pathway impact analysis, Figure S4: Schematic representation of metabolic alterations.
