# Supplementary material for: Metabolic Alterations in Pancreatic Cancer Progression
Source: Cancers (Basel). 2019 Dec 18;12(1):2. doi: 10.3390/cancers12010002 (PMC7016676; doi:10.3390/cancers12010002)
Supplement: Supplementary file 1 [file cancers-12-00002-s001.pdf]

Supplementary Figures

Fig. S1 Evaluation of purine pathway in pancreatic cancer models.

(a) Relative purine metabolite levels in 10, 15, and 25 weeks old KPC mice. (b) Relative purine metabolite levels in orthotopically implanted mice. (c) Relative purine metabolite levels in KPC cell lines. Abbreviations: IMP, inosine monophosphate; AMP, Adenosine 5'-monophosphate; dAMP, Deoxyadenosine 5'-monophosphate; ADP, adenosine 5'-diphosphate; ATP, adenosine 5'-triphosphate; GMP, Guanosine 5'-monophosphate; dGMP, Deoxyguanosine 5'-monophosphate; dGDP, Deoxyguanosine 5'-diphosphate. Data are represented as mean ± SEM. The bar charts in (a) and (b) were compared by Student's t-test $* \ p < 0.05$. Bar charts in (c) were compared by one-way ANOVA followed by Tukey's posthoc test # $p < 0.01$ and \$ $p < 0.001$.

Fig. S2 Evaluation of amino acids levels in pancreatic cancer models.

(a) Relative amino acids levels in 10, 15 and 25 weeks old KPC mice. (b) Relative amino acids levels in orthotopically implanted mice. (c) Relative amino acids levels in KPC cell lines. Data are represented as mean ± SEM. The bar charts in (a) and (b) were compared by Student's t test $*p < 0.05$, $**p < 0.01$, and $***p < 0.001$. Bar chart in (c) was compared by one-way ANOVA followed by Tukey's post-hoc test $*$ £ < 0.05 and $***$\$ < 0.001.

Fig. S3 Metabolic pathway impact analysis.

Representation of significantly upregulated metabolites analyzed by Metaboanalyst 3.0 in KCP mice tumors versus control mice pancreas.

Fig. S4 Schematic representation of metabolic alterations.

(a) Illustration of metabolic changes in 25 weeks-old mice, orthotopic implanted mice and cancer cell lines. Dashed arrows indicate the presence of one or more intermediate steps between the metabolites.

Figure S1

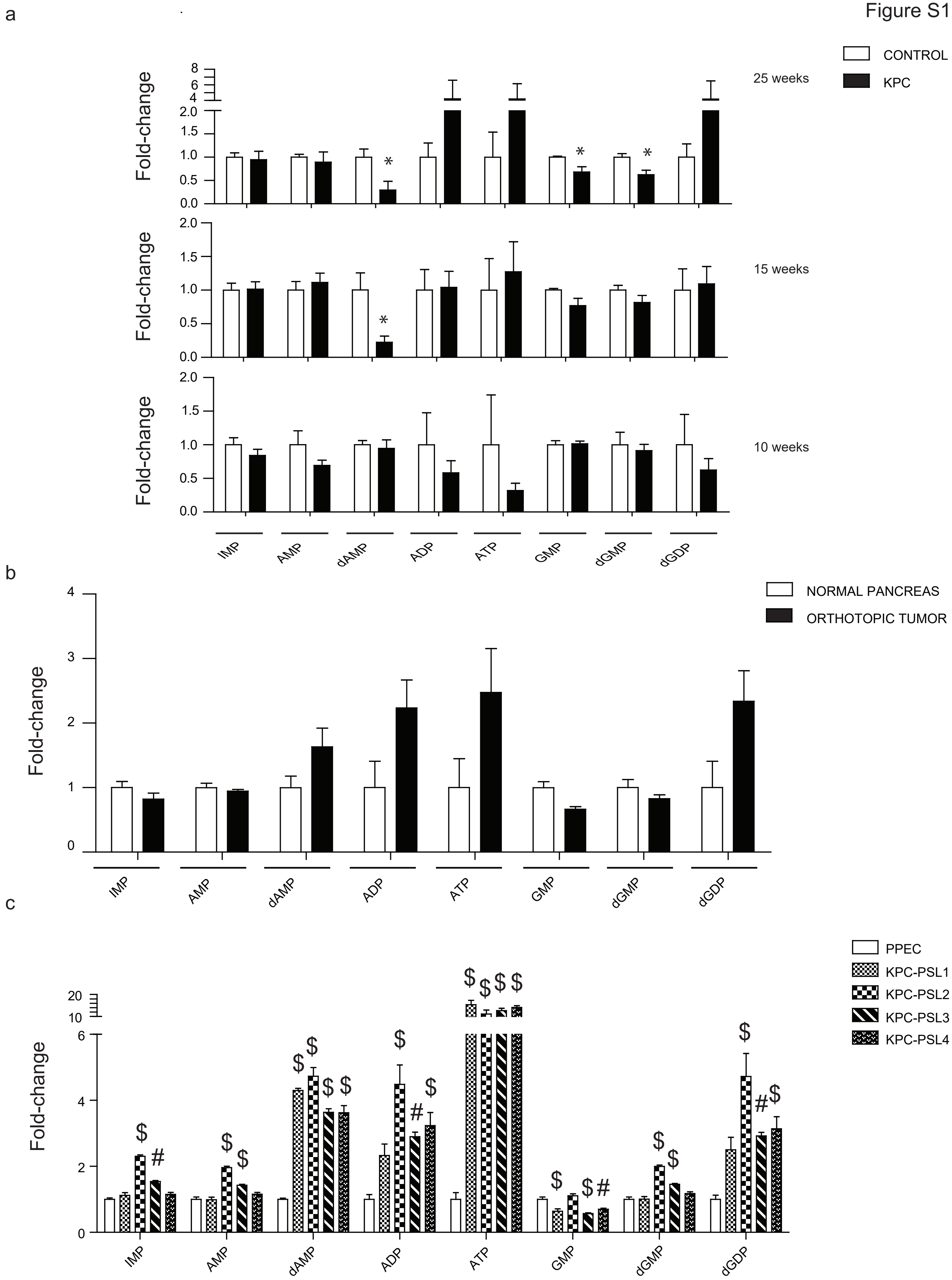

a
CONTROL
KPC
25 weeks
15 weeks
10 weeks
Fold-change
IMP
AMP
dAMP
ADP
ATP
GMP
dGMP
dGDP
b
NORMAL PANCREAS
ORTHOTOPIC TUMOR
Fold-change
IMP
AMP
dAMP
ADP
ATP
GMP
dGMP
dGDP
c
PPEC
KPC-PSL1
KPC-PSL2
KPC-PSL3
KPC-PSL4
Fold-change
IMP
AMP
dAMP
ADP
ATP
GMP
dGMP
dGDP

Figure S2

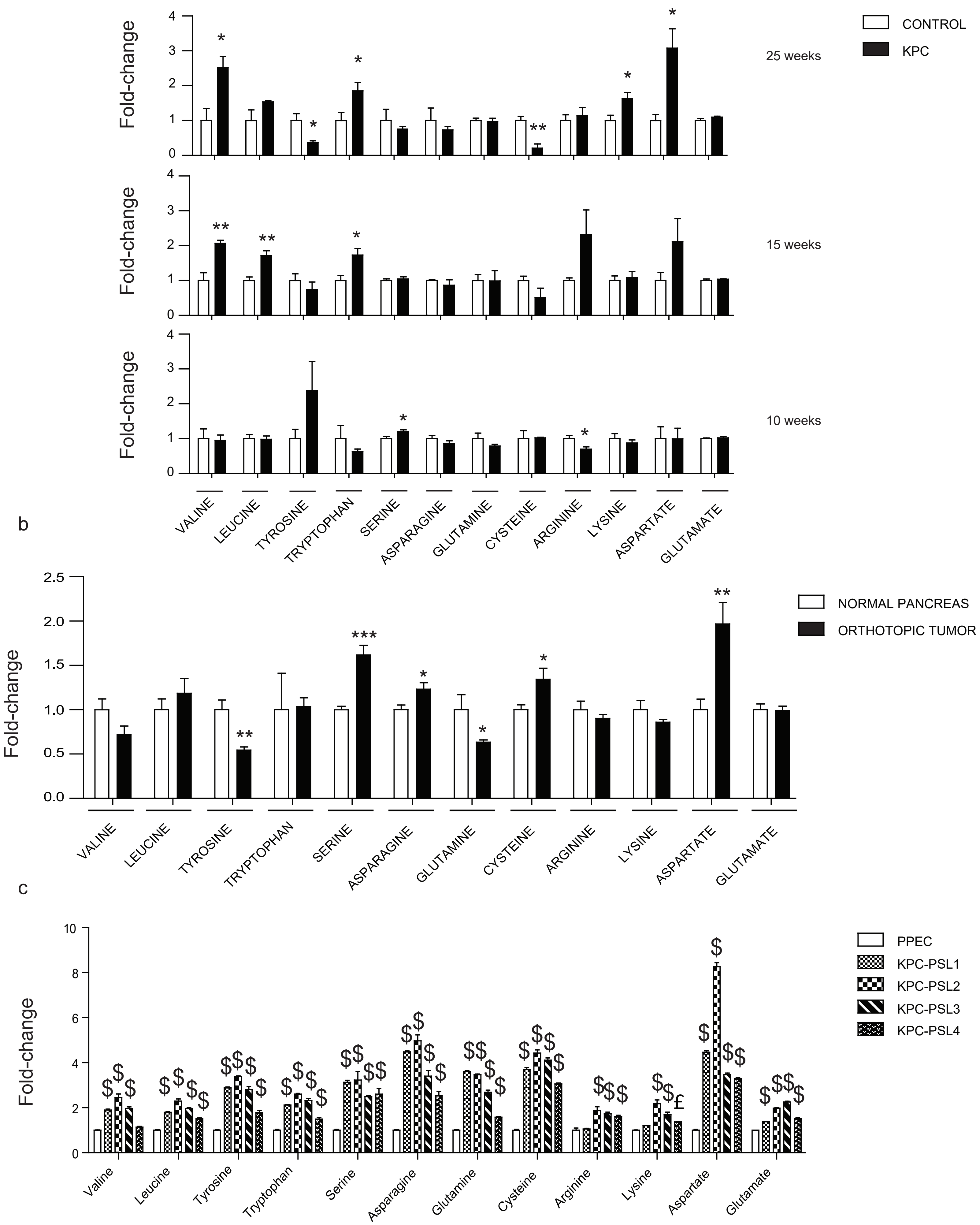
a
CONTROL
KPC
25 weeks
15 weeks
10 weeks
Fold-change
VALINE
LEUCINE
TYROSINE
TRYPTOPHAN
SERINE
ASPARAGINE
GLUTAMINE
CYSTEINE
ARGININE
LYSINE
ASPARTATE
GLUTAMATE
b
NORMAL PANCREAS
ORTHOTOPIC TUMOR
Fold-change
VALINE
LEUCINE
TYROSINE
TRYPTOPHAN
SERINE
ASPARAGINE
GLUTAMINE
CYSTEINE
ARGININE
LYSINE
ASPARTATE
GLUTAMATE
c
PPEC
KPC-PSL1
KPC-PSL2
KPC-PSL3
KPC-PSL4
Fold-change
Valine
Leucine
Tyrosine
Tryptophan
Serine
Asparagine
Glutamine
Cysteine
Arginine
Lysine
Aspartate
Glutamate

Figure S3

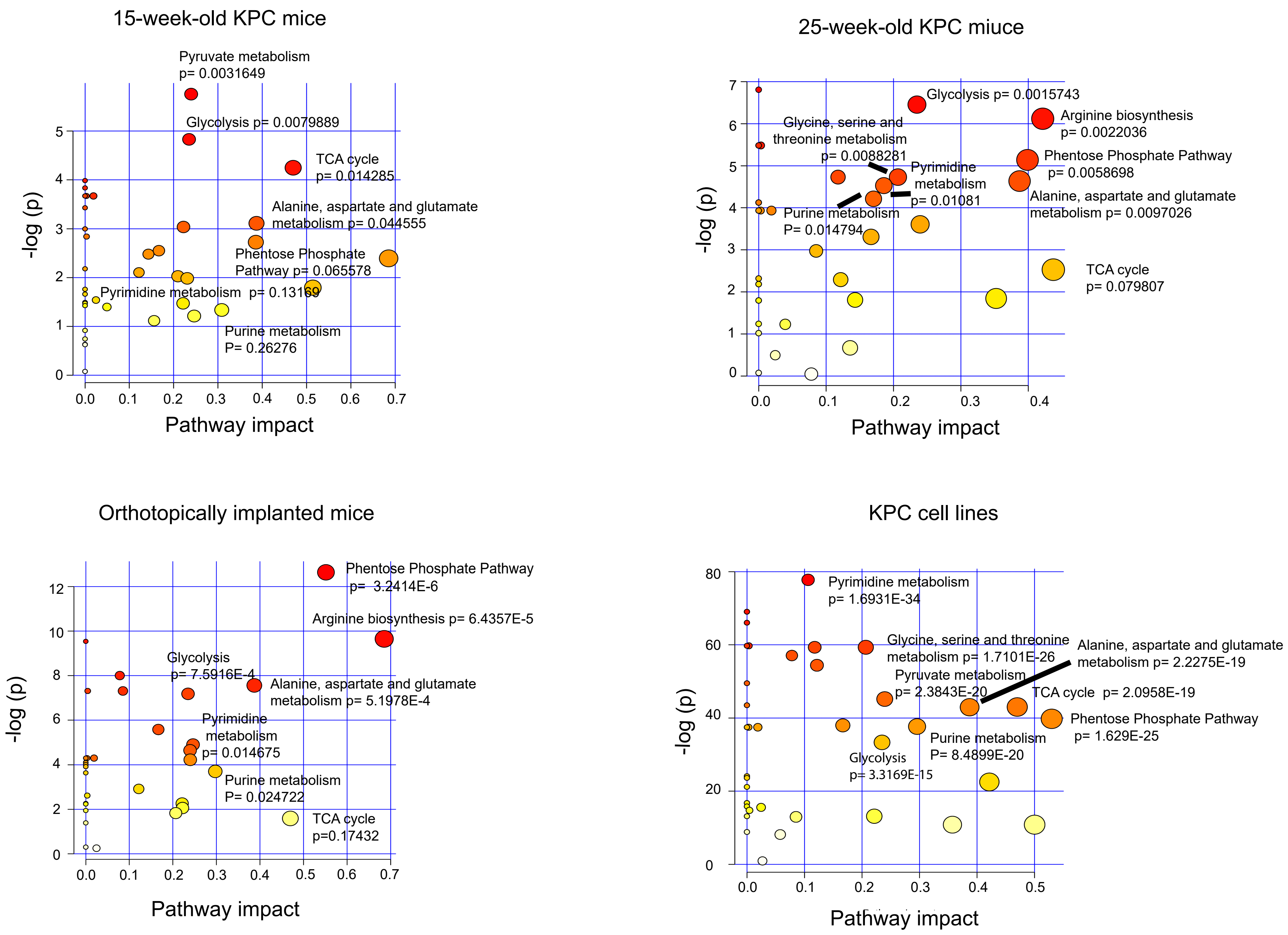

15-week-old KPC mice
Pyruvate metabolism p= 0.0031649
Glycolysis p= 0.0079889
TCA cycle p= 0.014285
Alanine, aspartate and glutamate metabolism p= 0.044555
Phentose Phosphate Pathway p= 0.065578
Pyrimidine metabolism p= 0.13169
Purine metabolism P= 0.26276
-log (p)
Pathway impact
25-week-old KPC miuce
Glycolysis p= 0.0015743
Arginine biosynthesis p= 0.0022036
Glycine, serine and threonine metabolism p= 0.0088281
Phentose Phosphate Pathway p= 0.0058698
Pyrimidine metabolism p= 0.01081
Alanine, aspartate and glutamate metabolism p= 0.0097026
Purine metabolism P= 0.014794
TCA cycle p= 0.079807
-log (p)
Pathway impact
Orthotopically implanted mice
Phentose Phosphate Pathway p= 3.2414E-6
Arginine biosynthesis p= 6.4357E-5
Glycolysis p= 7.5916E-4
Alanine, aspartate and glutamate metabolism p= 5.1978E-4
Pyrimidine metabolism p= 0.014675
Purine metabolism P= 0.024722
TCA cycle p=0.17432
-log (p)
Pathway impact
KPC cell lines
Pyrimidine metabolism p= 1.6931E-34
Glycine, serine and threonine metabolism p= 1.7101E-26
Alanine, aspartate and glutamate metabolism p= 2.2275E-19
Pyruvate metabolism p= 2.3843E-20
TCA cycle p= 2.0958E-19
Phentose Phosphate Pathway p= 1.629E-25
Purine metabolism P= 8.4899E-20
Glycolysis p= 3.3169E-15
-log (p)
Pathway impact

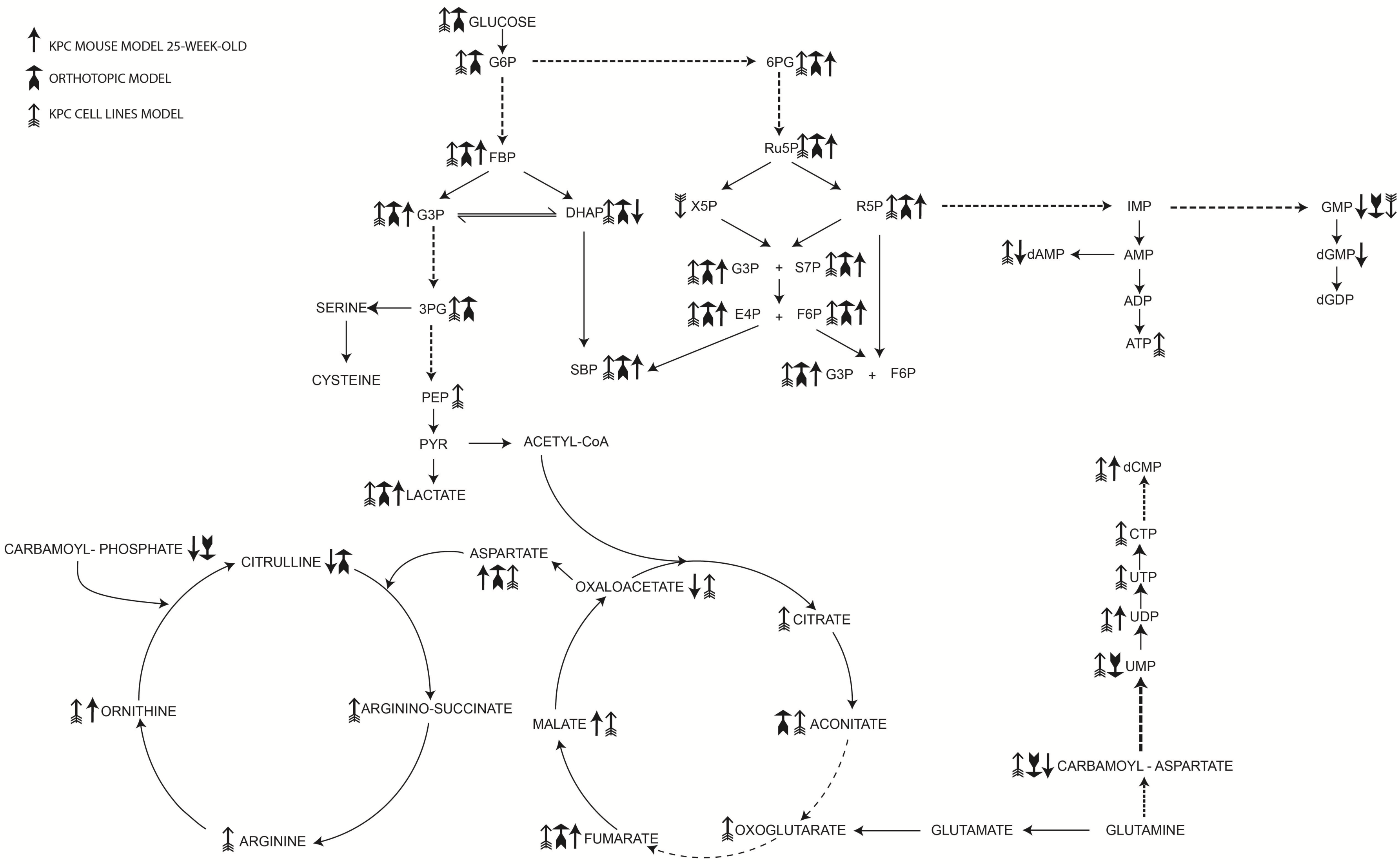

KPC MOUSE MODEL 25-WEEK-OLD
ORTHOTOPIC MODEL
KPC CELL LINES MODEL
GLUCOSE
G6P
6PG
FBP
Ru5P
G3P
DHAP
X5P
R5P
IMP
GMP
G3P
S7P
E4P
F6P
dAMP
AMP
dGMP
ADP
dGDP
ATP
SERINE
3PG
CYSTEINE
SBP
G3P
F6P
PEP
PYR
ACETYL-CoA
LACTATE
dCMP
CTP
UTP
UDP
UMP
CARBAMOYL- PHOSPHATE
CITRULLINE
ASPARTATE
OXALOACETATE
CITRATE
ORNITHINE
ARGININO-SUCCINATE
MALATE
ACONITATE
CARBAMOYL - ASPARTATE
ARGININE
FUMARATE
OXOGLUTARATE
GLUTAMATE
GLUTAMINE
